# Sixth sense in the deep-sea: the electrosensory system in ghost shark *Chimaera monstrosa*

**DOI:** 10.1038/s41598-022-14076-2

**Published:** 2022-06-14

**Authors:** Massimiliano Bottaro

**Affiliations:** Department of Integrative Marine Ecology (EMI), Genoa Marine Centre (GMC), Stazione Zoologica Anton Dohrn - Italian National Institute of Marine Biology, Ecology and Biotechnology, Villa del Principe, Piazza del Principe 4, 16126 Genoa, Italy

**Keywords:** Ecology, Zoology, Ichthyology, Marine biology

## Abstract

Animals that continually live in deep sea habitats face unique challenges and require adaptive specializations solutions in order to locate and identify food, predators, and conspecifics. The Ampullae of Lorenzini are specialized electroreceptors used by chondrichthyans for important biological functions. Ampullary organs of the ghost shark *Chimaera monstrosa*, a deep-sea species commonly captured as by-catch in the bottom trawl fishery, are here described for the first time using macroscopic, ultrastructural and histological approaches. The number of ampullary pores in *C. monstrosa* is about 700, distributed into the whole cephalic section of *C. monstrosa,* and organized in12 pore clusters and they are arranged into different configurations and form a distinct morphological pattern for this species, showing some anatomical peculiarities never described before in others cartilaginous fishes and may constitute an evolutionary adaptation of this ancient chondrichthyan species to the extreme environmental conditions of its deep sea niche.

## Introduction

The deep sea (> 200 m depth) includes about 95% of the world’s ocean volume and it is the greatest and not explored biome on the Earth^1^. The deep sea is close to total darkness and it has limited food resources and scarce mate opportunities^2^. Animals that live in the deep sea are faced with unique challenges and require sophisticated solutions in order to locate and identify food, predators, and each other. Chondrichthyans are important consumers in most marine ecosystems and show one of the most efficient array of sensory systems among marine fishes^3^, they are uncommon at depths below 3000 m, but a limited number of species live below 800 m^4–6^. Among the deep-sea cartilaginous fishes, the holocephalans (Chondrichthyes: Holocephali) are a small, ancient and poorly studied group of cartilaginous fishes that live worldwide in the deep environments^7,8^. Due to their regular occurrence in these extreme habitats and their phylogenetic position as sister and isolated group of elasmobranchs around 400 million years ago old, the sensory systems of chimaeroid fishes represent an interesting opportunity to better understand the evolution and the adaptations of chondrichthyans to the deep sea^9^. However, information on their senses, mainly on electroreception, is yet scarce at the moment, although chimaeroids have become increasingly threatened by expanding deep-sea fisheries^10,11^.

Like all others chondrichthyans, holocephalans have specialised ampullary electroreceptors, usually called Ampullae of Lorenzini, used for important biological functions, such as the detection of prey and/or possible predators, navigation, and mating^12,13^. Recent ampullary organ studies in elasmobranchs have focused on the relationships between morphological features, ecological niches, and the selective pressures that shaped the evolution of this sensory system in species with different life histories and behavioral repertoires^14–20^.

**Figure 1:**
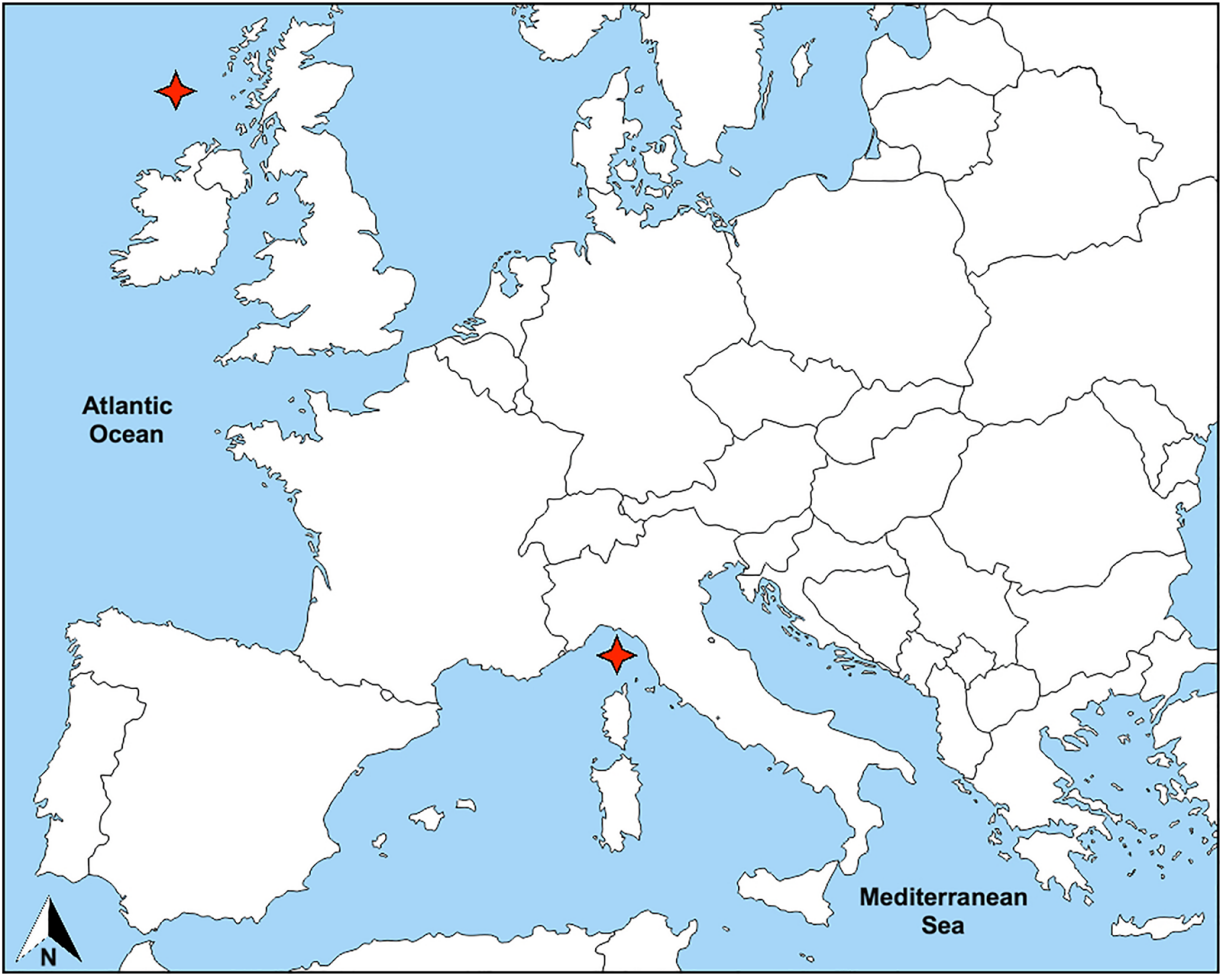
Sampling area of *Chimaera monstrosa* in the North-East Atlantic and in the Ligurian Sea (Western Mediterranean). Image modified from the original with permission (https://d-maps.com/carte.php?num_car=2232&lang=it).

Little electrosensory research has been done on holocephalans^21,22^, so we used an integrative approach to study the electroreceptors of the rabbit fish *Chimaera monstrosa* Linnaeus, 1758 (Holocephali; Chimaeridae). This species is distributed in the Eastern Atlantic and in the Mediterranean basin between −300 and −1000 m^23^, and it is commonly found in commercial bycatch of deep-sea bottom trawl fisheries^24,25^. *C. monstrosa* is an opportunistic predator feeding mainly on species associated with benthic community, such as crustaceans, molluscs, echinoderms and polychaetes^23,25–29^. The goals of this research are: (i) to describe the distribution, histological structure, and innervation of ampullary organs; (ii) to infer possible relationships between the anatomical structures and ecological patterns of *C. monstrosa;* and (iii) to emphasize the possible role of the sensory biology for the conservation of the deep-water cartilaginous fishes (Fig. 1).

## Results

### Ampullary pore distribution

**Figure 2:**
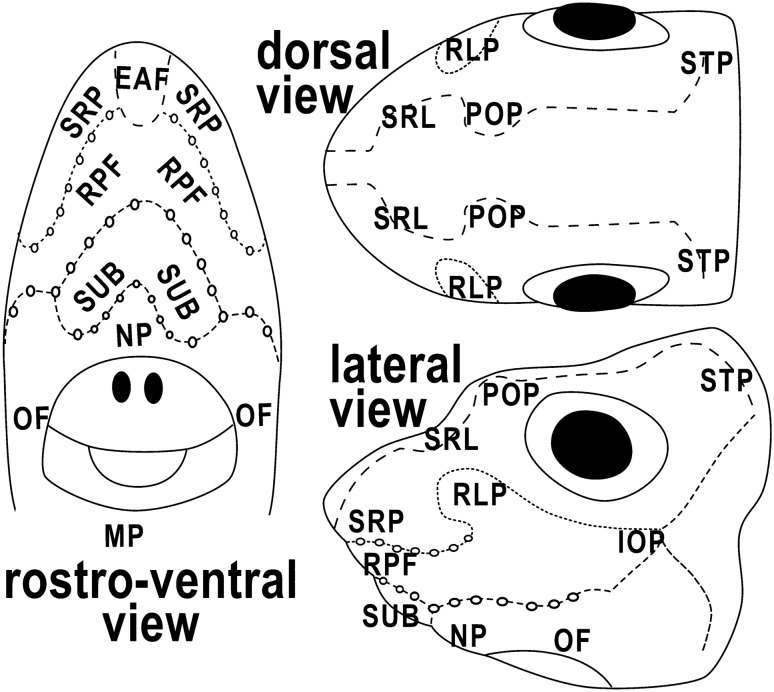
Gross anatomy of the head of the system of Ampullae of Lorenzini of *Chimaera monstrosa*. Lateral line canals are shown as dashed lines and circles. EAF: ethmoid cluster; IOP: infraorbital cluster; MP: mandibular cluster; NP: nasal cluster; OF: oral cluster; POP: preorbital cluster; RLP: rostrolateral cluster; RPF: rostral cluster; SRL: supra-rostrolateral cluster; SRP: suprarostral cluster; STP: supratemporal cluster; SUB: subrostral cluster.

The number of ampullary pores in our specimens of *Chimaera monstrosa* has an average of 702 (± 103). They are distributed in the entire cephalic portion. We recognized 12 types of pore clusters (Fig. 2): nine of them are double (one on each side of the head), while the ethmoid (EAF), nasal (NP) and mandibular (MP) are single clusters, that are settled in the medio-ventral part of the head. The total number and average pore size of every cluster type is summarized in Table 1.

**Table 1:** Count and measure of ampullary pores in *C. monstrosa.*

Name of the cluster	Average n. of pores for one cluster and relative standard deviation		No. of clusters	Average diameter of pores (mm) and relative standard deviation	
Ethmoid cl. (EAF)	22	6.4	1 (medial)	0.3	0.05
Infraorbital cl. (IOP)	10	1.1	2	1.2	0.20
Mandibular cl. (MP)	6	0.5	1 (medial)	0.6	0.11
Nasal cl. (NP)	38	3.3	1 (medial)	0.4	0.03
Oral cl. (OF)	18	1.8	2	0.3	0.09
Preorbital cl. (POP)	14	1.7	2	0.4	0.09
Rostrolateral cl. (RLP)	11	2.4	2	0.5	0.08
Rostral cl. (RPF)	32	5.3	2	0.5	0.20
Supra-rostrolateral cl. (SRL)	88	16.0	2	0.2	0.03
Suprarostral cl. (SRP)	55	5.9	2	0.4	0.09
Supratemporal cl. (STP)	10	0.4	2	0.9	0.14
Subrostral cl. (SUB)	79	12.2	2	0.4	0.07

The average total number of pore (702 ± 103) was calculated by adding the average number for each medial cluster and the double average number for each double cluster.

**Figure 3:**
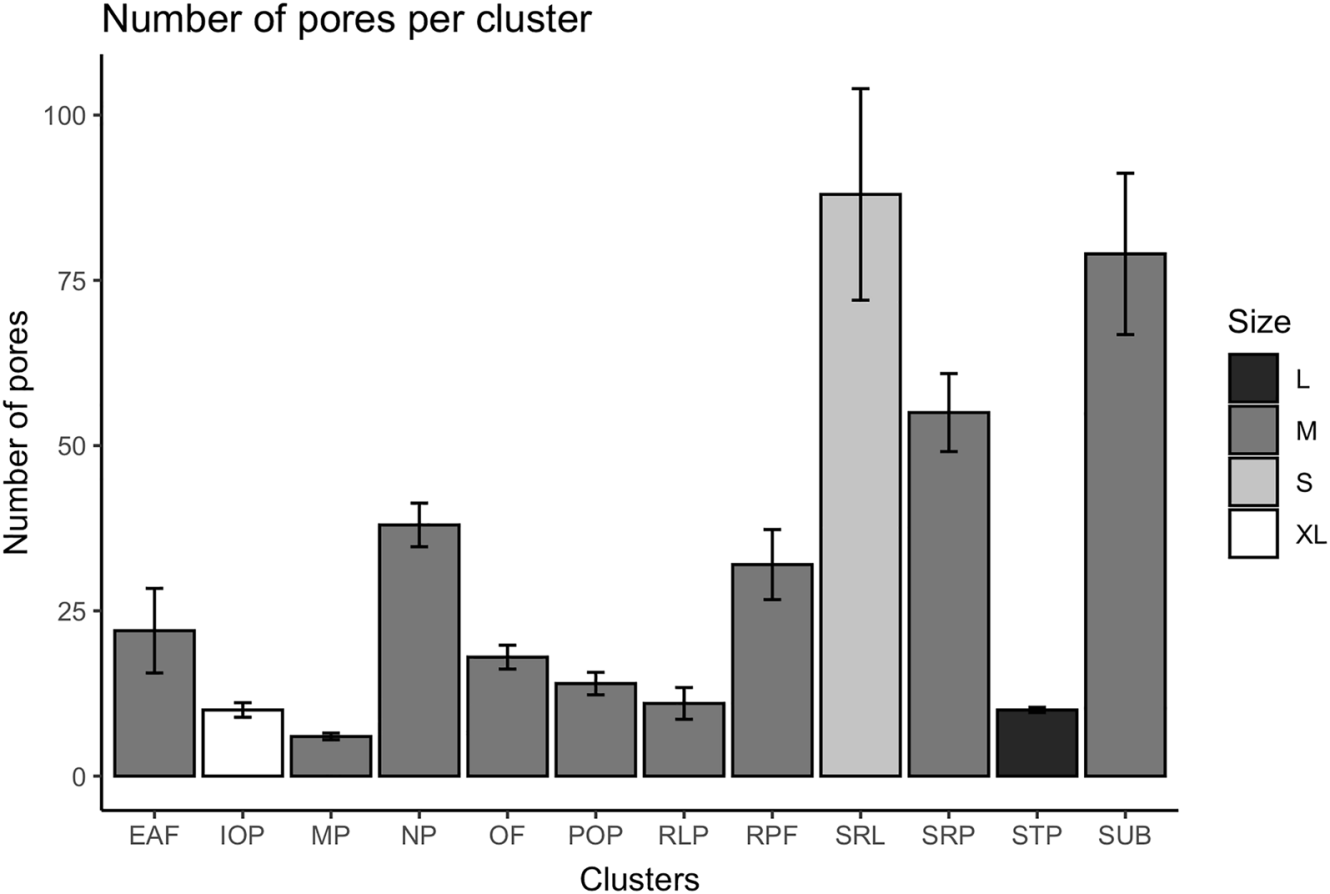
Histogram of the average number of pores for each cluster. For the cluster which are double (present in the same position on both sides of the head) the number of pores here represented is for only one cluster. Four shade of grey indicate the size of the pore. XL: extra-large sized pore (average size larger than 1 mm); L: large sized pores (average size between 0.9 and 1.0 mm); M: medium sized pores (average size between 0.3 and 0.6 mm); S: small sized pores (average size smaller than 0.3 mm). The choice of these different classes of size depends on the ANOVA and Tukey test performed (see Fig. 3), which indicate a statistically significant difference of the average size of IOP (1.2 mm average pore diameter), SRL (0.2 mm average pore diameter), and STP (0.9 mm average pore diameter) clusters among them and among all other clusters. EAF: ethmoid cluster; IOP: infraorbital cluster; MP: mandibular cluster; NP: nasal cluster; OF: oral cluster; POP: preorbital cluster; RLP: rostrolateral cluster; RPF: rostral cluster; SRL: supra-rostrolateral cluster; SRP: suprarostral cluster; STP: supratemporal cluster; SUB: subrostral cluster.
- size—the range diameter is between 0.2 and 12 mm and the various clusters reveal difference also in the dimension of the pores summarized in Table 1 and Fig. 4.

In particular:

- density—the pores are mainly concentrated in 3 clusters (suprarostral STP, supra-rostrolateral SRL, and subrostral SUB) which include almost the 65% of the whole ampullary pores and which have a medium or small pore diameter (Table 1 and Fig. 3);

**Figure 4:**
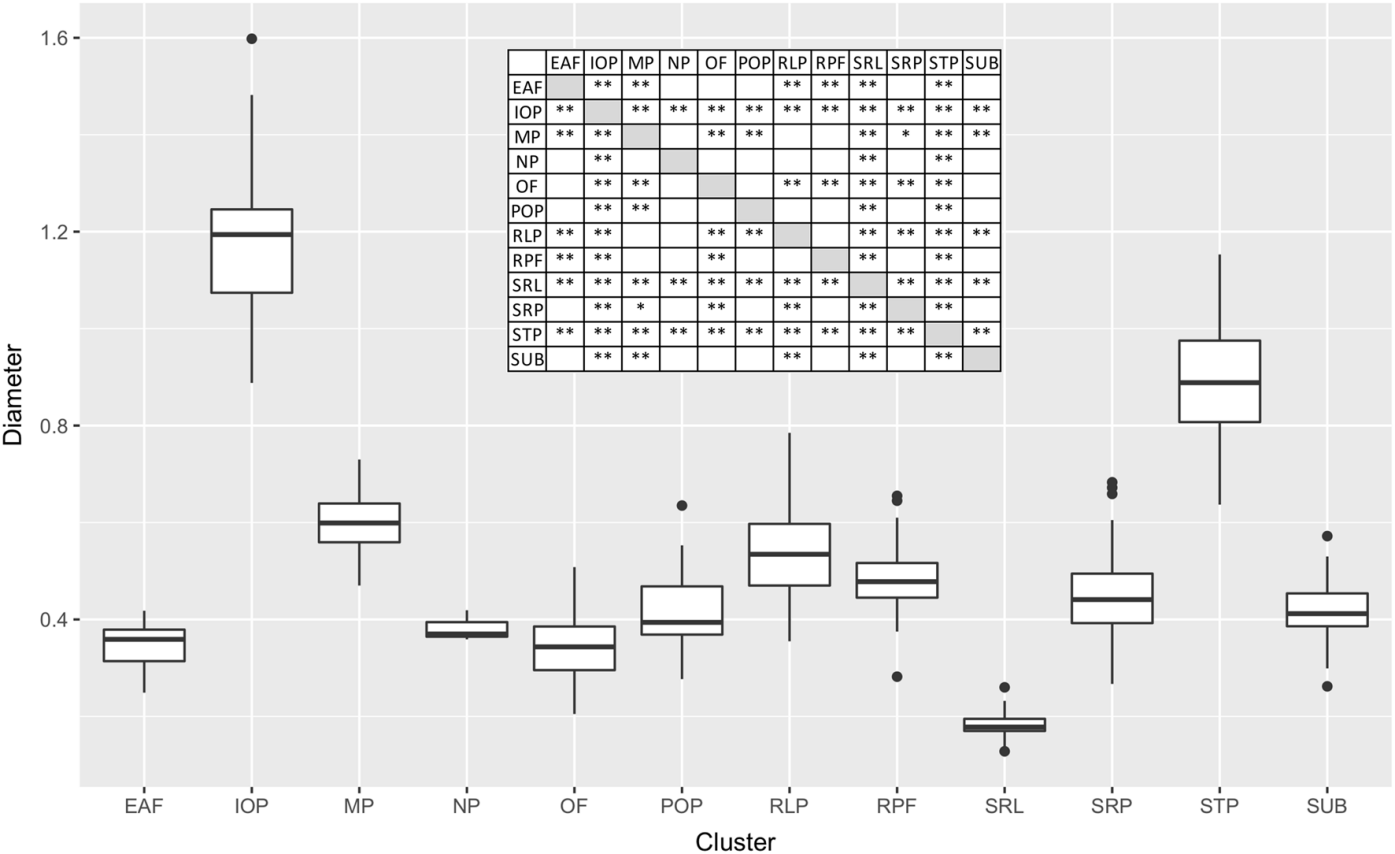
Box-plot of the measured pore size for each cluster. The diameter is in mm. In the table the results from the Tukey test **: *p* value < 0.05; *p-value < 0.1. Three clusters have an average pore size statistically different from all other cluster (IOP, SRL, and STP). EAF: ethmoid cluster; IOP: infraorbital cluster; MP: mandibular cluster; NP: nasal cluster; OF: oral cluster; POP: preorbital cluster; RLP: rostrolateral cluster; RPF: rostral cluster; SRL: supra-rostrolateral cluster; SRP: suprarostral cluster; STP: supratemporal cluster; SUB: subrostral cluster.

### Gross morphology

**Figure 5:**
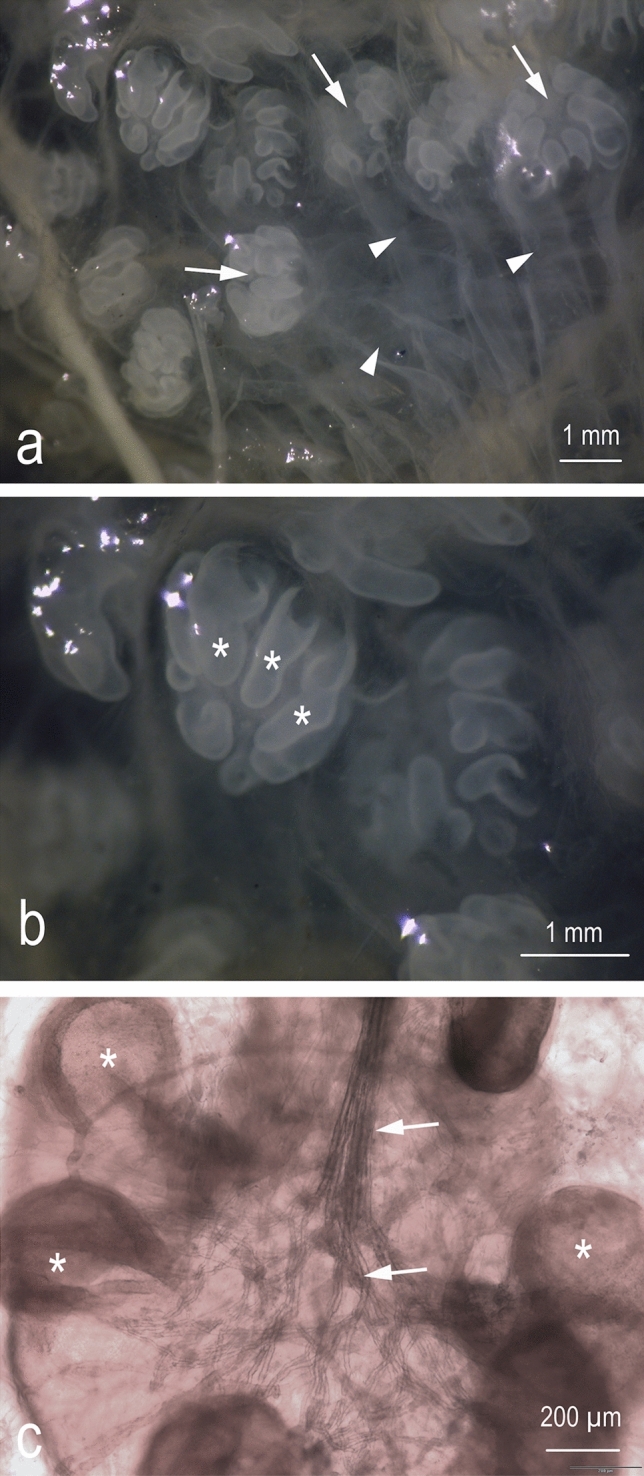
Ampullary electroreceptors of *C. monstrosa*. (**a**) stereomicroscope photograph of a group of Ampullae. The canals are slightly visible (arrowheads) and ends in the ampullary part (arrows). (**b**) detail of figure (**a**). The ampullary part is subdivided in chambers (asterisks). c) A whole ampulla through the light microscope. The transmitted light allows to observe the nerve fibers (arrows) in the center of the ampulla, among the chambers (asterisks).

Observations of the gross morphology show that in both sexes the electroreceptive organs of *C. monstrosa* are organized into clusters and consist of a canal and dilated ampullary portion that are filled with a gel and surrounded by connective tissue (Fig. 5a). The ampullary portion of the sensory organ is formed by eight or nine alveolar sensory chambers (Fig. 5b). The proximal end of the ampullary portion is connected to one afferent nerve (Fig. 5c).

### Ultrastructure

**Figure 6:**
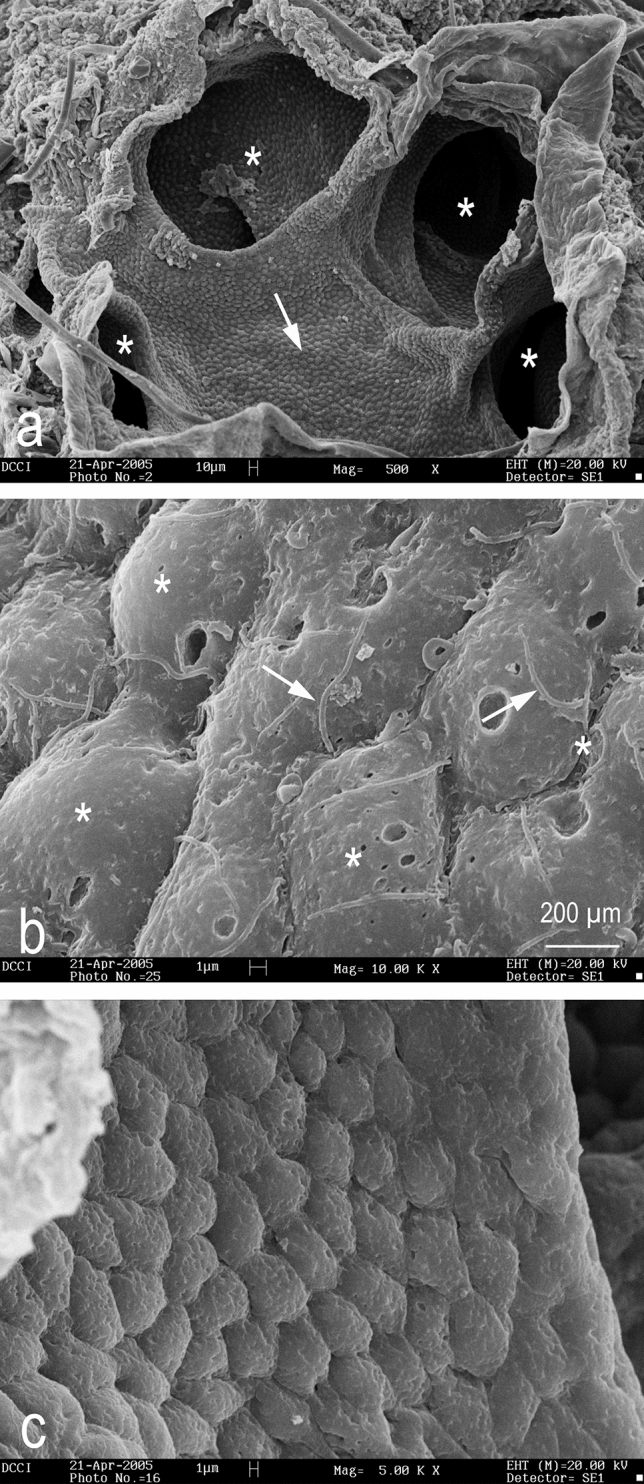
SEM micrograph of ampullary electroreceptors of *C. monstrosa*. (**a**) The chambers (asterisks) and a central plate (arrow) are visible; (**b**) the sensory epithelium in alveoli; (**c**) the epithelium covering the canal. Among the supporting cells (asterisks), the sensory cilia of the sensory cells are visible (arrows).

SEM observations allowed us to analyze the superficial structure of the alveolar portion of the Ampullae of Lorenzini and the ampullary canal, revealing no differences among the sexes. The alveolar portion of the chambers converge into a common area (Fig. 6a) and are composed of a single columnar epithelium that contains supporting and sensory cells (Fig. 6b), However, the canals are distinguished from the alveolus by their composition of simple squamous epithelium (Fig. 6c).

### Histology

**Figure 7:**
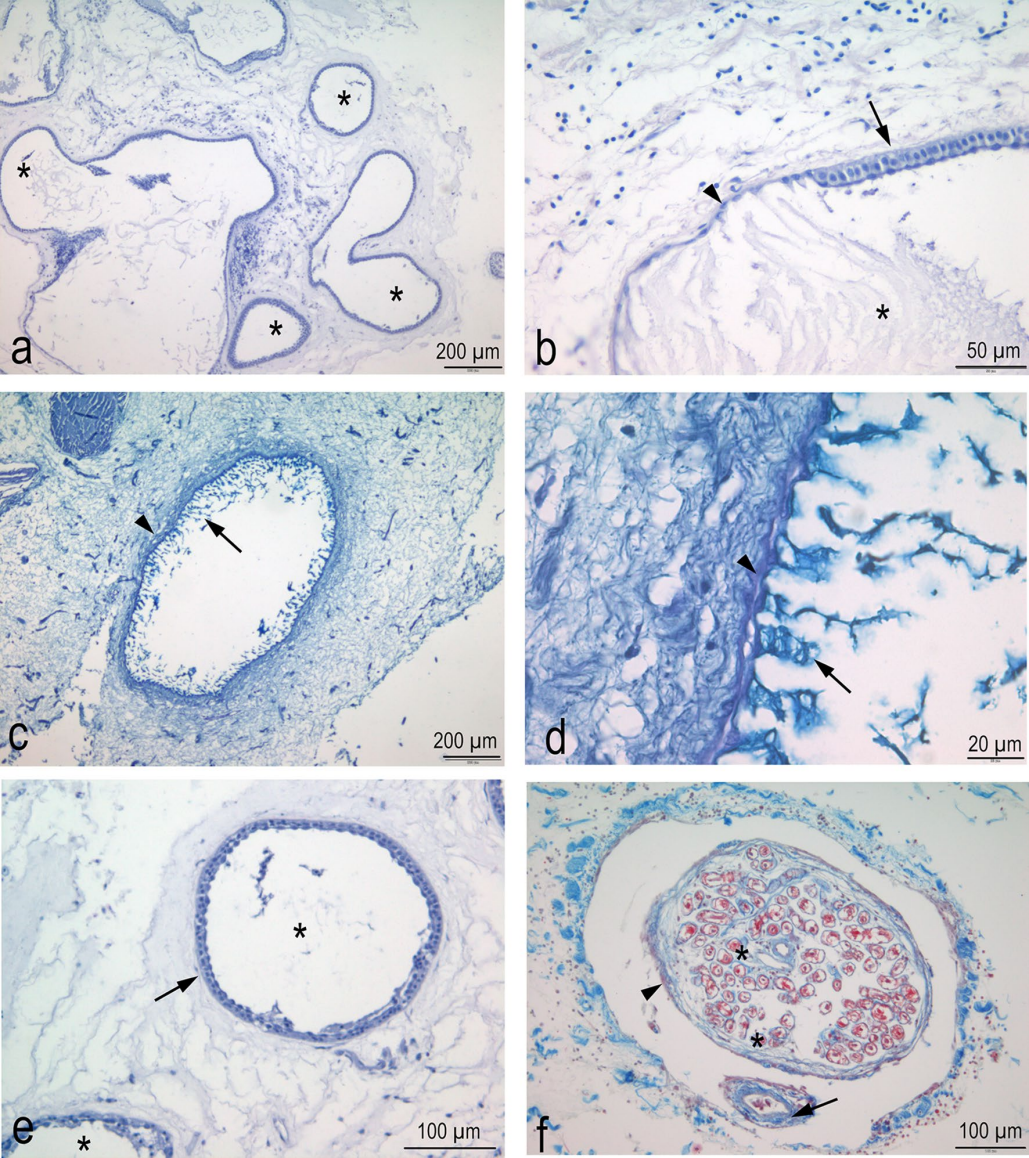
Histological sections of ampullary electroreceptors of *C. monstrosa*. (**a**) Hematoxylin–Eosin staining. A loose connective tissue surrounds the canal and the chambers (asterisks). (**b**) Hematoxylin–Eosin staining. The sensory epithelium (arrow) is thicker than the epithelium of the canal (arrowhead) and the border between the two type of epithelium is made up few cells with an intermediate morphology. Although not stained, the gel substance, secreted by the canal lining cells, is visible (asterisk). (**c**, **d**) Alcian-PAS staining. The canal epithelium (arrowheads) secretes an Alcian-positive substance (arrows) in the canal lumen. (**e**) Hematoxylin–Eosin staining. Alveoli (asterisk) are lined up by a sensory epithelium (arrow). (**f**) Masson’s trichrome staining. In the connective tissue surrounding the Ampullae, the nerve fibers are visible. They are enveloped in perineurium (arrowhead) and run along with blood vessels (arrow). Each axon is enveloped in endoneurium (asterisk).

Histological analysis confirms the general organization of the ampullary organs and the histological structure of the canal and the alveolar portion (organized in chambers), both surrounded by connective tissue composed mainly of collagen (Fig. 7a). There is a clear histological separation between the epithelia of these two parts divided by a medial zone of cuboidal-shape cells (Fig. 7b).

**Figure 8:**
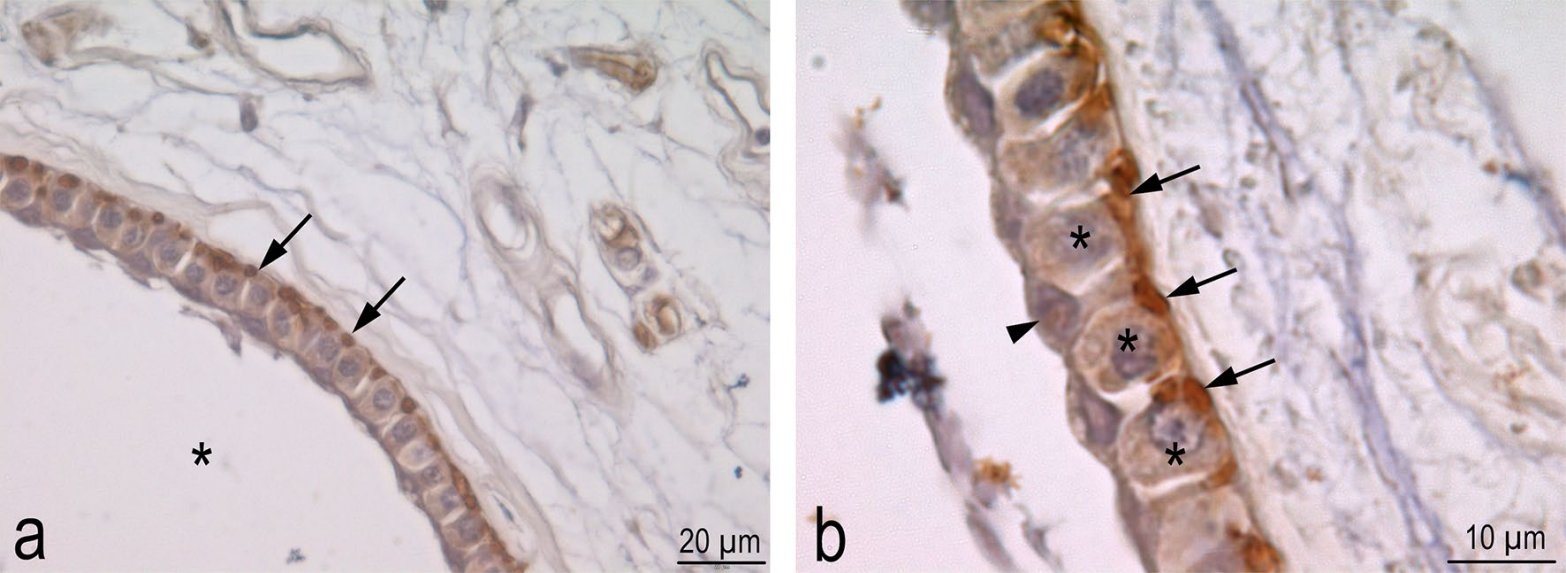
Histological sections of ampullary electroreceptors of *C. monstrosa*. Na^+^/K^+^-ATPase immunohistochemisty (brown) and Hematoxylin. a) The sensory epithelium lining the chambers (asterisk) presents basally Na/K-ATPase-like immunoreactivity (arrows). b) The epithelium is made up by at least two types of cells, the supporting cells (arrowhead) and the sensory cells. The Na/K-ATPase-like immunoreactivity in the basal part of sensory cells could highlight the nerve terminals.

The canal of the Ampullae of Lorenzini is, in fact, covered by a simple squamous epithelium (Fig. 7c), also presenting mucous flattened cells showing Alcian positive gel protrusions (Fig. 7d); while, on the contrary, the ampullary portion appears to consist of a simple columnar epithelium, formed by supporting and sensory cells (Figs. 7e, 8). The central part of the ampullary portion is formed by the same columnar epithelium. Bundles of afferent nerve fibers reach the base of each alveolar portion, below the central part (Fig. 7f) and nerve terminals were observed at the base of the sensory cells only (Fig. 8).

## Discussion

While teleosts have successfully colonised the deep sea, chondrichthyans are uncommon^5^. The species who live there rely on their sensory systems to gather information about their environment and to guide their behaviour^30^. Despite species of the order Holocephali regularly dwell in deep sea habitats, little is yet known about their biology and ecology^7^, and the information regarding their sensory systems are very limited^10,31,32^. Here we describe for the first time the Ampullae of Lorenzini in the rabbit fish *Chimaera monstrosa*, providing morphological details and attempting to clarify their functional role. Although electroreception in shallow-water have been the subject of a number of studies^12^, which have started to investigate also the correlation between the morphology and the lifestyle^18^, this sensory system has been more rarely investigated in deep sea species^11^, even less so in the Holocephalans^10,21,22^.

Compared to elasmobranchs^12^, and according to previous data on the other genus of chimaerids, *Hydrolagus*^21^, *C. monstrosa* shows a lower number of electroreceptive ampullary pores. This might be related to the feeding habits of this species: it is an opportunistic suction-feeder that actively searches the prey near the sea bottom^24,26,27,33^, and then uses its tooth plates to crush the food^29^. In fact, relatively few pores and low electrosensory resolution are mainly observed in cartilaginous fishes which feed with an indiscriminate suction or ram-feeding methods of prey capture^12^.

The location of pores determines the spatial representation and direction of the electrosensory field around the head^34^. In *C. monstrosa*, more pores are located on the anterior frontal sides of the head and ventrally near the mouth, while the pores with the largest diameter are located dorsoventral to the eyes. In this area we describe the supra-rostrolateral (SRL) cluster, a group of ampullary pores never described before in Holocephalans. Most of the pore clusters fields are located facing forward, which allows *C. monstrosa* to detect potential prey that swims along the bottom. The highest density of pores is found near the mouth because the primary function of electroreception is to detect prey and correctly position the subterminal mouth during the final strike on prey^35^. Therefore, pore number and location correlate with the foraging strategy^36–38^. The positions of the clusters with fewer but largest pores might allow the fish to detect possible the presence of possible predators during navigation and prey searching: *C. monstrosa* is actively preyed upon by larger deep-sea dogfishes^28,39^. This functional hypothesis is supported by the fact that chondrichthyans feeding on the sea bottom, like *C. monstrosa*, have limited routes of escape, being partially blocked by the substrate. They usually position their anti-predatory countermeasures, such as tail, fin spines and additional electrosensory pores, along the dorsal and posterior body surfaces^12^.

Ampullary organs have been classified into three different types based on the size and the length of the canals: (1) macro-ampullae, (2) micro-ampullae, and (3) mini-ampullae^40^. *C. monstrosa* presents the so-called “macro-Ampullae”, characterized by large, visible pores and long canals. As the others cartilaginous fishes, electroreceptors in *C. monstrosa* are embedded in a gelatinous substance and organized in clusters containing several functional sensory units, the ampullary organs. The gross anatomy of the ampullary portion it is not organized in alveoli usually arranged in a grape-like formation^12^, but it is constituted by numerous independent finger-shaped sacs. This suggests a morphological pattern typical for *C. monstros*a or for chimaerids^21^. This anatomical feature might enhance the sensory surface in relation of deep sea environment. It was previously observed that both the number of alveolar chambers and the overall size of the Ampullae significantly increase with depth, and species inhabiting deeper regions of the water column possess higher numbers of receptor structures, in order to improve feeding capability in a relatively prey-poor environment^15,16^. In addition, unlike what is observed in some derivate elasmobranchs, like the catshark *Scyliorhinus canicula*^41^, in *C. monstrosa* the size of ampullary organs and the number of finger-shaped sacs are the same in both sexes, and it might be related with the more primitive phylogenetic position of this species^42^.

The histological organization and innervation of the ampullary organs of *C. monstrosa* are similar to those already described for some coastal benthic elasmobranchs^20,43–45^. The presence of very abundant Alcian positive gel both in the canal and in the ampullary portion suggests a very important role of this mucopolysaccharidic substance also for *C. monstrosa*. It has been demonstrated, in fact, that ampullary gel plays a fundamental role as a semiconductor with temperature-dependent conductivity and thermoelectric behavior, as a simple ionic conductor with the same electrical properties as the surrounding seawater, and as proton-conductor as well^46–49^. The extreme scarcity of light and the limited probability to find food typical of deep sea environments require adaptations of the chondrichthyan sensory systems^35^, which might also include enhanced efficiency in the transmission of stimuli mediate by this mucopolysaccharidic substance. Another histological characteristic is that the central part of the ampullary portion seems to be constituted by the same sensory epithelium as the alveolar finger-shaped sacs. This contrasts with what was observed in others chondrichthyans, where the central region appears to be formed by typical cells, called “brush cells”^19,40,44^.

Variations in alveolar morphology exist within the different ampullary groups, and they have been classified into five types, based on alveolar arrangement: “single-alveolate”, “multi-alveolate”, “branched alveolate”, “centrum cap” and “club-shaped”^39^. The majority of sharks, including deep sea species, possess the “centrum cap” type^15^. The ampullary structure observed in *C. monstrosa* is similar to the so called “central cap” type already suggested for other species^44^. This organization is beneficial for detecting a variety of stimuli, therefore representing a possible evolutionary adaptive improvement of this phylogenetically conserved sensory system.

There are over 1300 extant species of cartilaginous fishes^50^ that arose sometime during the group 400 million years evolution, with the main living families having first appeared sometime between the Permian and Jurassic Periods^51^. Currently, they occupy a very broad range of habitats worldwide, and play a key role in the functioning of ecosystems^52^. Under selective pressure, the chondrichthyan sensory systems, including olfaction and electroreception, have been differentially optimised to function in various habitats^33,53^: in particular, recent researches revealed how electrosensory system is adapted to suit the lifestyle or environmental niche of a species, also through discrete molecular and biophysical modifications^54^. The electroreception in *C. monstrosa* reveals here some anatomical peculiarities never described before in others cartilaginous fishes, which might constitute an evolutionary adaptation of this ancient chondrichthyan species to the extreme environmental conditions of its deep sea niche^18^. In this view, Holocephali could be a very interesting model for future comparisons to the study of chondrichthyan ampullary system because : (1) it is a small and phylogenetically isolated group constituted by different closely related species, occupying different deep sea habitats and with also different ecological features^8,54^, which makes them a very interesting group in which to examine the relationship between structure and function; (2) they reside mostly in deep-sea, a light-limited environment where non-visual senses, like electroreception is most likely to be important^55–57^.

In this framework, future research should verify if some deep-water elasmobranchs have a similar pattern to *C. monstrosa* or if it is unique to Chimaerids, in order to clarify more the evolutionary and ecological role of this peculiar sensory system.

## Methods

All experimental protocols followed the recommendations of the Committee for the Animal Welfare of the Stazione Zoologica Anton Dohrn (https://www.szn.it/index.php/en/who-we-are/organization/committee-for-the-animal-welfare). Moreover, all methods were carried out in accordance with relevant guidelines and regulations of the European Union.

Adult specimens of *Chimaera monstrosa* were collected as by-catch in the Ligurian Sea (North-West Mediterranean Sea) and in the North-East Atlantic (Fig. 1) Ocean by professional bottom trawlers operating at the depth range of −600 and −800 m. They were brought already dead on board and the taxonomical identification was rapidly carried out on board^22^. After anesthesia with 0,01% MS-222 (tricainemethanesulfonate; Argent, Redmond, WA, USA; dilution 1:1000 in sea water), the rostral parts of their heads were excised immediately and fixed in 4% p-formaldehyde solution in phosphate buffered saline, pH 7.4 (PBS). Alternatively, some specimens were frozen at -30° C. In the laboratory, ampullary clusters were removed from the fixed rostral part of heads, isolated and prepared for macroscopical analysis or microscopical observations for both sexes; frozen specimens were used for ampullary pore distribution description.

### Ampullary pore distribution

After defrosting, heads were severed from the specimens in the transverse plane at the first gill because no ampullary pores are located caudal to this position. Pores were counted for each cluster. The number and diameter of pores were measured by direct observation through a dissecting microscope or via ImageJ^58^ for areas of high pore density. The average number of pores (+ /− SD) were calculated across all specimens. Pore diameter was measured in multiple images for each specimen and an average diameter (+ /- SD) was calculated. The nomenclature used to describe ampullary pore distribution following Didier^21^. The average pore diameters from different clusters were tested using ANOVA and Tukey HSD test. The graphical representations of the data were performed using R software^59^ and the ggplot2 package^60^.

### Macroscopical methods

After washing in PBS, ampullary clusters and single Ampullae were observed and examined by a Zeiss Stemi 2000 C stereomicroscope. Images were acquired by a CellPad E (TiEsseLab S.r.l., Italy).

### Scanning electron microscope methods

Samples for scanning electron microscopy (SEM) were osmium postfixed, dehydrated through a graded ethanol series, mounted on stubs, sputter coated with gold and examined by SEM Leo Stereoscan 440 (LEO Electron Microscopy Ltd.).

### Histomorphological, histochemical and immunohistochemical methods

After washing in PBS, the samples were dehydrated, Paraplast embedded (Bioptica, Italy) and 6 µm sectioned. Dewaxed and rehydrated sections were alternatively haematoxylin–eosin stained or treated with Alcian blue pH 2,5- PAS method for carbohydrate detection. In order to highlight the presence of nerve endings, Na^+^/K^+^ATPase immunoreactivity was assessed by pre-treating the slides with Bovine Serum Albumine to block non-specific antibody binding. Next a mouse monoclonal antibodyspecific for the α subunit of chicken Na^+^/K^+^ATPase (α5, supernatant, 0.9 mg/ml, DSHB, USA) and then an anti-mouse FITC conjugated antiserum (1:400 in PBS, Agilent DAKO, USA) was used. Negative controls were performed by omission of the primary antibody or by using the NS1 hybridoma culture supernatant (DSHB, USA) as primary antibody. Alternatively, the immunoreactivity was detected using the Agilent Dako EnVision + Kit with HRP in order to increase sensitivity and to minimize non-specific background staining. In this case the incubation with the primary antibody was preceded by the using the blocking reagents for endogenous peroxidase and nuclei were counterstained using Hematoxylin.

The sections were examined with an Olympus BX60 microscope (light and epi-fluorescence microscope) and visualized through the Color-View Camera (Olympus, Japan). The images were acquired and analysed through the software AnalySIS (Soft Imaging System, USA).
